# Overexpression of PLK1 relieved the myocardial ischemia-reperfusion injury of rats through inducing the mitophagy and regulating the p-AMPK/FUNDC1 axis

**DOI:** 10.1080/21655979.2021.1938500

**Published:** 2021-06-11

**Authors:** Shan Mao, Shuning Tian, Xianghong Luo, Ming Zhou, Zheng Cao, Ji Li

**Affiliations:** a Department of Cardiology, Taihe Hospital, Hubei University of Medicine, Shiyan City, Hubei Province, China; b Department of Anesthesiology, Jiangmen Central Hospital Affiliated Jiangmen Hospital of Sun Yat-sen University, Jiangmen City, Guangdong Province, China; c Department of Anesthesiology, Taihe Hospital, Hubei University of Medicine, Shiyan City, Hubei Province, China; d Department of Cardiovascular Internal Medicine, The Second Affiliated Hospital of Guizhou Medical University, Kaili City, Guizhou Province, China

**Keywords:** Myocardial ischemia-reperfusion injury, PLK1, p-ampk, FUNDC1, mitophagy

## Abstract

Myocardial cell injury caused by myocardial ischemia and reperfusion is one of the main causes of the occurrence and development of heart disease. Recent study has shown that inducing mitophagy of cardiomyocytes is a crucial method to alleviate ischemia-reperfusion injury. While, Polo-like kinase 1 (PLK1) can induce the mitophagy of breast cancer cells. Moreover, PLK1 was able to promote the expression of p-AMPK and FUNDC1, which are the protective factors for myocardium. Therefore, the mouse model of ischemia/reperfusion was established and the effect of PLK1 on ischemia reperfusion induced myocardial damage was investigated. The PLK1 was overexpressed in H9c2 cells and rat model of ischemia/reperfusion. Ischemia reperfusion inhibited the expression of PLK1. While overexpression of PLK1 relieved the myocardial infarction and myocardium apoptosis through inducing mitophagy in rats model of ischemia reperfusion. In vitro, the H9c2 cells overexpressing the PLK1 were treated with the hypoxia and reoxygenation and the apoptosis, survival rate and expression of mitophagy-related proteins of H9c2 cells were detected using the flow cytometry, CCK-8 assay and western blotting. The results reveled that overexpression of PLK1 alleviated the hypoxia and reoxygenation induced apoptosis of H9c2 cells and promoted the expression of mitophagy-related proteins. In addition, enhanced PLK1 expression promoted the expression of p-AMPK and FUNDC1 in H9c2 cells. However, the inhibition of FUNDC1 abolished the positive effect of PLK1 on H9c2 cells mentioned above. In conclusion, PLK1 alleviated the ischemia reperfusion induced myocardial damage by inducing the mitophagy in a p-AMPK/FUNDC1 signaling dependent pathway.

## Graphical Abstract

**Figure:**
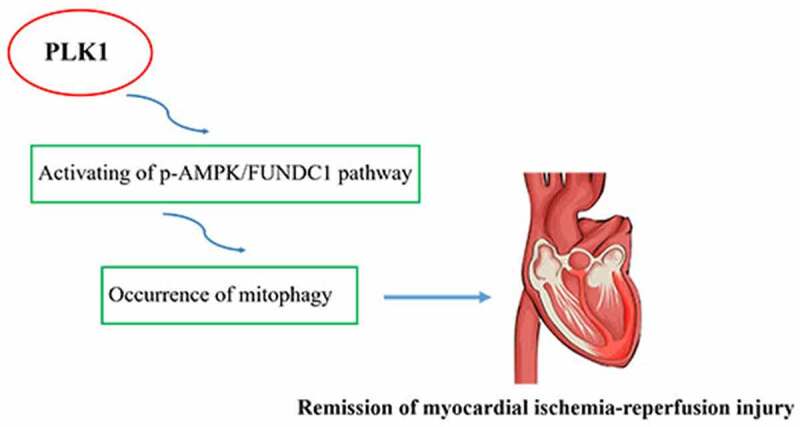

## Introduction

Myocardial infarction (MI) is the main cause of high disability and mortality in cardiovascular disease patients worldwide. The mechanism of myocardial repair after MI is very complicated [1]. Currently, the treatment for myocardial infarction just only relieve the symptoms, but cannot solve the problem of myocardial cell loss [2]. Some studies have confirmed that myocardial cell death is a critical cause of ventricular remodeling, cardiac insufficiency and arrhythmia after myocardial infarction. Whereas myocardial ischemia induced mitochondrial dysfunction can result in the death of myocardial cells [3,4]. Mitochondria are organelles which can produce the reactive oxygen species (ROS), while more than ten times ROS will be generated in damaged mitochondria compared to normal mitochondria. Large amounts of ROS production can directly induce the damage of mitochondrial proteins and DNA, aggravate the dysfunction of mitochondrial, and form the vicious circle [5]. Studies have also shown that proper mitophagy will protect the organism from ischemia-reperfusion injury [6,7]. Therefore, regulating mitophagy of cardiomyocytes is a potential strategy for treating the myocardial infarction.

Polo-like kinase (PLK) is a highly conserved serine/threonine kinase, which plays a key role in regulating cell cycle progression and coordinating cell division [8]. Polo-like kinase 1 (PLK1) is the most distinctive member in PLK families, which can promote the proliferation of multiple types of cells through regulating mitosis. Furthermore, PLK is also expressed in tumor tissues and is capable of suppress the apoptosis of tumor cells [9,10]. PLK1 participated in the process of neuronal autophagy and decreased the expression of PLK1 will indirectly inhibit mitophagy of breast cancer cells [11,12]. The expression of PLK1 also plays a critical role in the proliferation of cardiomyocytes during heart regeneration [13]. Therefore, we speculated that PLK1 may be involved in myocardial protection and mitophagy. However, the efficacy and mechanism of PLK1 affecting ischemia-induced myocardial injury remain elusive.

Some studies have found that PLK1 promoted the progression of mitosis through regulating the activity of AMPK [14,15]. Nevertheless, activating AMPK can protect cardiomyocytes from apoptosis [16]. Moreover, some study found that activation of AMPK increased mitophagy and fusion by up-regulating OPA1 expression, therefore relieving the myocardial ischemic injury [17]. Furthermore, AMPK can adjust the expression of FUNDC in multiple types of cells. For example, the expression of mitochondrial receptor FUNDC1 in cells was increased after electrical pulse stimulation activating the AMPK pathway, thereby inducing the mitophagy [18]. FUNDC1 is a new type of mitochondrial receptor, which plays a protective role in heart disease [13]. Activating of FUNDC1 can increase the mitochondrial activity of heart cells after ischemia-reperfusion injury, inhibit mitochondrial apoptosis, reduce oxidative stress in cardiomyocytes, and maintain mitochondrial membrane potential and ATP production [19]. Importantly, FUNDC1 can also induce mitophagy for protecting mitochondria [20]. Some work suggested that FUNDC1 can maintain mitochondrial function by inducing the mitophagy of cardiomyocytes, thereby protecting myocardial damage caused by ischemia and reperfusion [21]. However, inhibiting of FUNDC1-mediated mitophagy will aggravate the heart damage caused by ischemia-reperfusion injury [22]. Therefore, we speculated that PLK1 may protect the myocardium from ischemia-reperfusion injury through inducing mitophagy by regulating the AMPK/FUNDC1signaling pathway, and ultimately. Here, the study will clarify the mechanism of myocardial ischemia reperfusion, which will provide new strategies for the clinical treatment of myocardial ischemia reperfusion.

## Material and methods

### Animal assays

Twelve SD rats was obtained from the laboratory animal center of Chinese Academy of Sciences (Shanghai, China). These rats were divided into two groups (sham and ischemia reperfusion group, six rats per group). The rats model of ischemia reperfusion was established according the referenced method [23]. The hearts of rats were collected and used for the subsequent experiments after the rats were killed. Furthermore, another 24 rats with ischemia reperfusion were divided into four groups (sham+Ad-NC, sham+Ad-PLK1, IR+Ad-NC and IR+Ad-PLK1, six rats per group) and adenovirus overexpressing PLK1 and corresponding negative control (obtained from the Genechem, Shanghai, China) were injected into the rats. Similarly, the hearts and serum of these rats were collected for following analysis. The animal assays were examined and approved by the ethics review committee of Taihe Hospital, Hubei University of Medicine.

### Staining of heart tissues

The ventricles were sliced into sections after sacrificing the rats. Followingly, the sections were stained with 1% triphenyltetrazolium chloride (Beyotime, China) and 2% evans blue stain (Beyotime, China) were for 20 minutes at 37°C. Then, the heart tissues were observed and photographed using the microscope (Olympus, Japan). The infarcted area of the heart tissues was measured with the ImageJ software (National Institutes of Health, USA).

### Cell culture and treatment

Rat myocardial cell line (H9c2 cells) was purchased from the ATCC (Manassas, VA, USA). These cells were cultured with RPMI1640 medium supplemented with 10% fetal bovine serum (Gibco, USA) at 37°C a humidified atmosphere with 5% CO_2_. To simulate the physiological environment of ischemia reperfusion of myocardial cells, the cells of H/R group were cultured with glucose-free balanced salt solution in 95% N_2_, 5% CO_2_, and 37°C environment for 2 hours. Then, the culture medium was replaced with complete medium to mimic the reperfusion process. H9c2 cells were transfected with adenovirus for overexpressing PLK1and lentivirus for knocking down, FUNDC1 and corresponding negative control were also established . Polybrene (Genechem, China) was used for enhancing the transfection efficacy and FUNDC1 inhibitor (Dorsomorphin, also named as compound C, Thermo Fisher Scientific, USA) was used for additional treatment of these cells.

### ELISA assays

Serum of rats and culture medium of the cells were collected for the ELISA assays. Rat LDH ELISA kit (Abcam, ab183367), human GSH ELISA kit (Abcam, ab19534), human GPX ELISA kit (Abcam, ab195174) and human SOD ELISA kit (Abcam, ab178012) were used for the detection of the expression of these factors in serum of rats or culture medium of H9c2 cells. The ATP production in H9c2 cells was also detected using ELISA assay with commercial kits according to instruction [24].

### CCK-8 assays

Cells were plated into the 96 well plates (2 × 10^3^ cells per hole). Then, cells were treated with the CCK-8 (Dojindo, Japan) for 2 hours at 37°C and the absorbance was measured with the spectrophotometer (Thermo Fisher Scientific, USA) [25].

### Apoptosis assays

The apoptosis of cells was detected with Tunel staining and flow cytometry using commercial Tunel staining kits (Beyotime,Nantong, China). Following staining, PBS was used for washing cells for three times. Then, cells were for incubated with Annexin V and PI (Beyotime, China) for 30 minutes. Finally, the apoptosis of cells was analyzed with the flow cytometry [26].

### RT-PCR

Total RNA was extracted with the trizol (Thermo Fisher Scientific, USA) and reverse transcription of the RNA was performed with reverse transcription kit (Takara, Japan). Then, the cDNA was amplified with the ABI7500 system (Thermo Fisher Scientific, USA). 2^−ΔΔCt^ method was used for analyzing the results [27]. The primers used in this research were PLK1 forward primer 5ʹ-UAUUCAUUCUUCUUGAUCCGG-3ʹ reverse primer 5ʹ-UUAGUCGACAUGUAAACCA-3ʹ GAPDH forward primer 5ʹ-GGAGCGAGATCCCTCCAAAAT-3ʹ reverse primer 5ʹ-GGCTGTTGTCATACTTCTCATGG-3ʹ.

### Western blotting

Total protein was extracted with the RIPA buffer (Beyotime, USA). The protein samples of mitochondria were extracted with the commercial kits (Invitrogen, USA). BCA method was used for determining the concentration of protein. After that, these proteins were separated with 10% SDS-PAGE gel (Beyotime, China) and transferred to PVDF membranes (Millipore, USA). Then, proteins were blocked with the BSA (Beyotime, China) and incubated with the primary antibodies at room temperature for 2 hours followed by an incubation with the secondary antibodies for 2 hours at room temperature. The primary antibodies used in this research were PLK1 (Abcam, ab189139), Bax (Abcam, ab32503), Bcl-2 (Abcam, ab32124), cleaved caspase3 (Abcam, ab32042), LC3 I (Abcam, ab63817), LC3 II (Abcam, ab51520), Beclin1 (Abcam, ab207612), p-AMPK (Abcam, ab133448), AMPK (Abcam, ab32047), FUNDC1 (Abcam, ab224722) and β-actin (Abcam, ab8226). The primary antibodies were diluted with the BSA with the ratio (1:1000). Finally, the Pierce Western Blotting Substrate (Thermo Fisher Scientific, USA) was used for the detection of the immunoreactive signals. The quantification of bands was performed with the ImageJ (National Institutes of Health, USA) [28].

### Immunofluorescence assays

H9c2 cells were plated on the glass slide. and fixed with 4% paraformaldehyde (Beyotime, China) after adhesion to slide. Then, cells were treated with 0.5% Triton X-100 (Beyotime, China) and incubated with the primary antibodies for 2 hours at room temperature. The antibodies used in this research were Tom20 (Abcam, ab186735) and LAMP1 (Abcam, ab24170). Followingly, these cells were incubated with the secondary antibodies (Anti-mouse IgG, CST, #4408 for Tom20 and Anti-rabbit IgG, CST, #8889 for LAMP1) for 2 hours in dark room. Finally, the DAPI (Invitrogen, USA) was used for staining the cell nucleus of H9c2 cells and the fluorescence was observed with the microscope (Olympus, Japan) [29].

### Statistical analysis

All the data were analyzed with the Graphpad Prism 6.0. Data was expressed as mean ± SD (N = 3). The comparison between two groups was performed with the student’s t test. *p* < 0.05 was considered as statistically significant difference.

## Results

In this study, we speculated that the expression of PLK1 could relieve the ischemia reperfusion induced myocardial injury by activating mitophagy. The effect of PLK1 on the development of myocardial ischemia-reperfusion injury and the potential molecular mechanism was investigated, which provides a new target for treating ischemia reperfusion induced myocardial damage .

### Ischemia reperfusion injury repressed the expression of Polo-like kinase 1 (PLK1)

**Figure 1.**
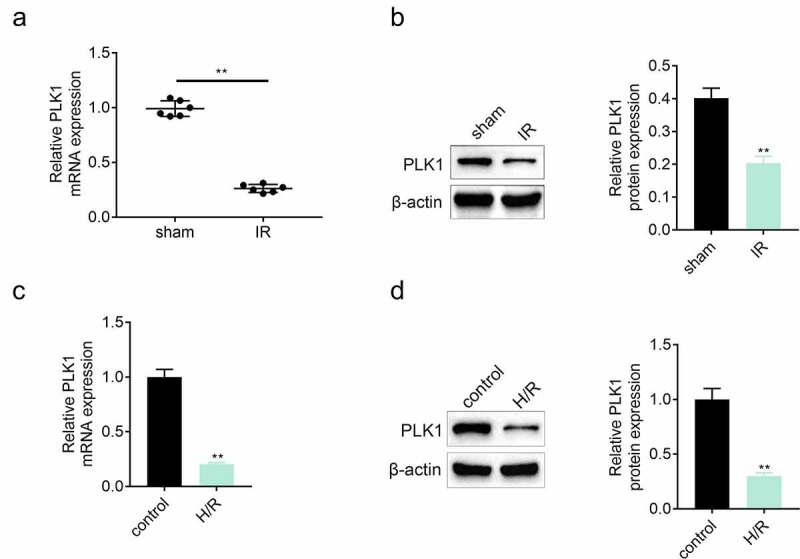
**The expression of PLK1 was suppressed in myocardium of rats suffered from the ischemia reperfusion**. (a, b) the mRNA and protein expression of PLK1 in myocardium of rats detected by RT-PCR and western blotting. (c, d) the expression of PLK1 in H9c2 cells determined by RT-PCR and western blotting. **p < 0.01

The expression of PLK1 in myocardium was detected by the western blotting and RT-PCR after establishing the model of ischemia reperfusion. Results (Figure 1a and Figure 1b) showed that the expression of PLK1 was suppressed in myocardium of rats suffered from ischemia reperfusion injury. In *in-vitro* assay, the expression of PLK1 in H9c2 cells was also inhibited after the stimulation of hypoxia and reoxygenation (Figure 1c and Figure 1d).

### Overexpression of Polo-like kinase 1 (PLK1) relieved the ischemia reperfusion induced myocardial infarction of rats

**Figure 2.**
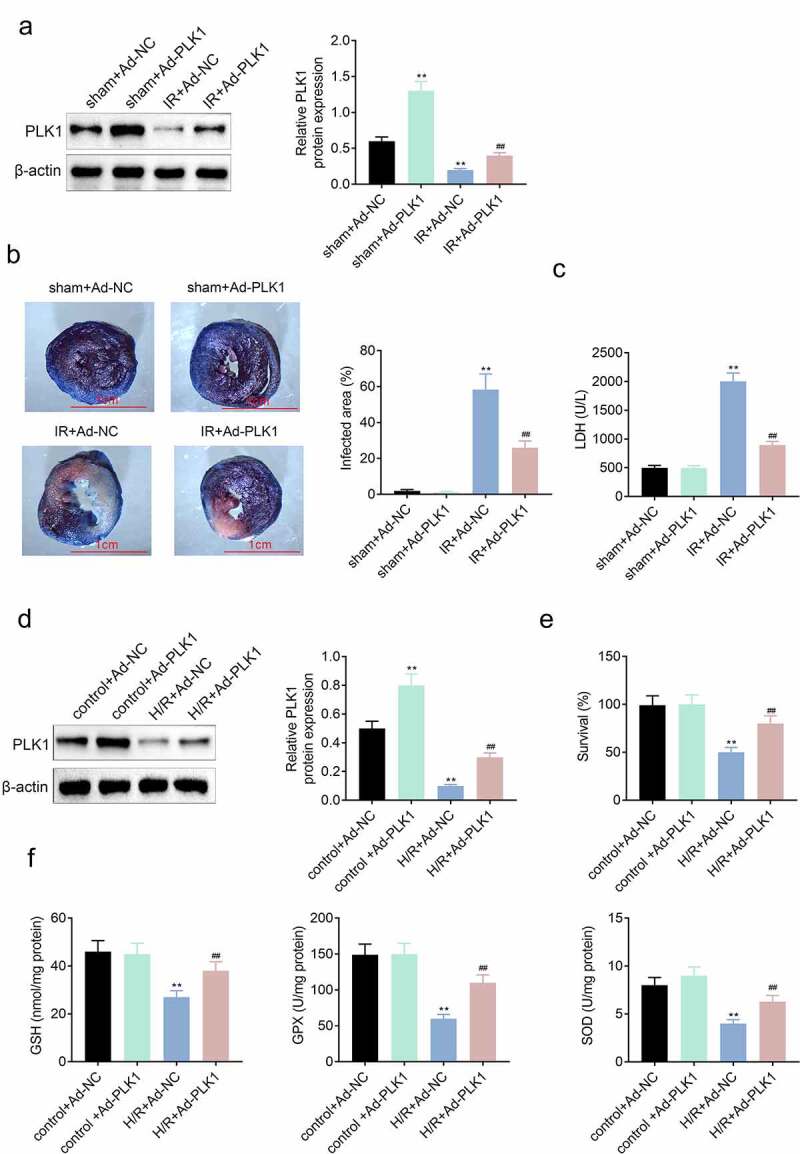
**Overexpression of PLK1 relieved the ischemia reperfusion induced myocardial infarction of rats**. (a) Western blot analysis of the expression of PLK1 in myocardium of rats determined by. (b) the myocardial infarction area detected by evans blue staining. (c) the levels of LDH in the serum of rats determined by ELISA. (d) Western blot analysis of the expression of PLK1 in H9c2 cells. (e) the survival rates of H9c2 cells detected by CCK-8 assay. (f) the levels of GSH, GPX and SOD in H9c2 cells detected by ELISA. ## p < 0.01 IR+Ad-PLK1 *vs* IR+Ad-NC, **p < 0.01 sham+Ad-NC vs IR+Ad-NC

The PLK1 was overexpressed in H9c2 cells through transfecting adenovirus. As shown in Figure 2a, the expression of PLK1 was enhanced in H9c2 cells in sham plus Ad-PLK1group. While, the triphenyltetrazolium chloride and evans blue staining results revealed that both the infarcted myocardial area and the level of LDH in serum of ischemia reperfusion rats were significantly reduced after enhancing the overexpression of PLK1 in rats (Figure 2b & 2c) . The *in-vitro* results (Figure 2d) also showed that the expression of PLK1 was increased in H9c2 cells transfected with PLK1 adenovirus. The CCK-8 assay results showed that overexpression of PLK1 enhanced the survival rates of H9c2 cells stimulated with the hypoxia and reoxygenation (Figure 2e). In addition, the release of GSH, GPX and SOD was decreased in H9c2 cell by stimulation with hypoxia and reoxygenation but which was reversed by the overexpression of PLK1.

### Overexpression of polo-like kinase 1 (PLK1) relieved the ischemia reperfusion induced apoptosis of myocardium of rats

**Figure 3.**
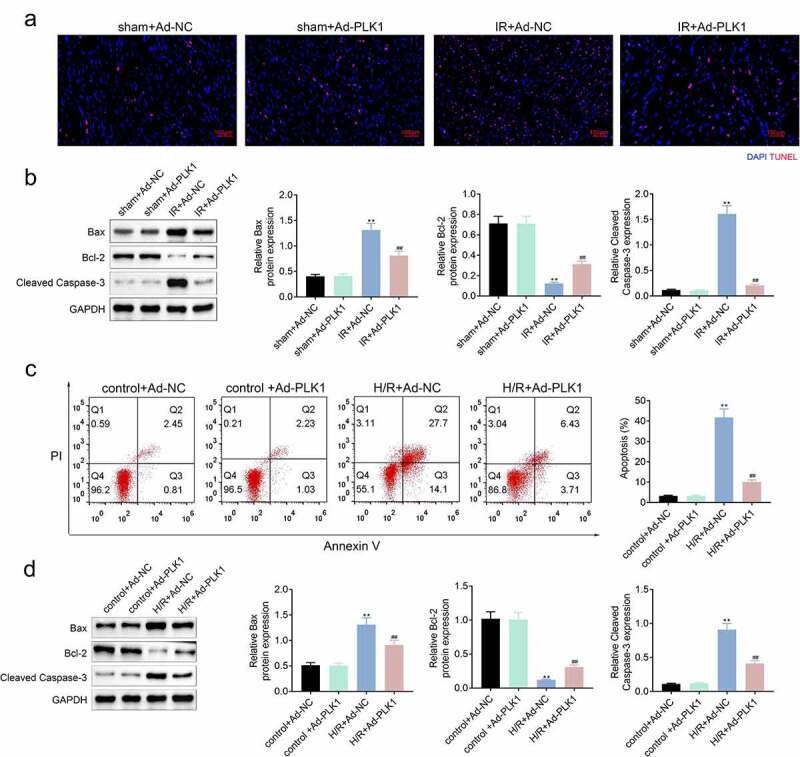
**Overexpression of PLK1 relieved the ischemia reperfusion induced apoptosis of myocardium of rats**. (a) the apoptosis of myocardium detected by tunel staining. (b) Western blot analysis of the expression of apoptosis related proteins in myocardium. (c) flow cytometry analysis of the apoptosis of H9c2 cells by. (d) Western blot analysis of the expression of apoptosis related proteins in H9c2 cells. ##p < 0.01 IR+Ad-PLK1 *vs* IR+Ad-NC, **p < 0.01 sham+Ad-NC vs IR+Ad-NC

Tunel assay was used for the detection of the apoptosis of myocardium tissues of rats. As shown in Figure 3a, overexpression of PLK1 relieved the ischemia reperfusion induced apoptosis of myocardium tissues of rats. Furthermore, the expression of Bcl-2 was also enhanced, while the Bax and cleaved caspase3 expression were decreased after overexpressing PLK1 (Figure 3b). Similarly, the apoptosis of cells and apoptosis-related proteins in H9c2 cells suffered from hypoxia and reoxygenation were also decreased after the overexpression of PLK1 (Figure 3c&3d).

### Overexpression of polo-like kinase 1 (PLK1) induced the mitophagy and protected the mitochondria of H9c2 cells

**Figure 4.**
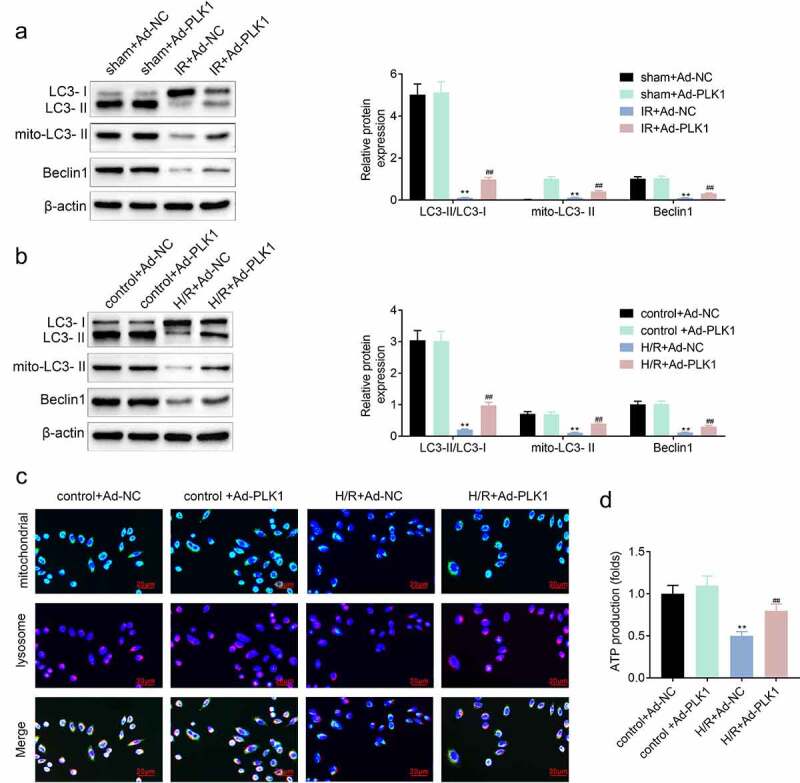
**Overexpression of PLK1 induced the mitophagy of myocardium of rats suffered from the ischemia reperfusion**. (a, b) Western blot analysis of the expression of mitophagy related proteins in myocardium of rats and H9c2 cells. (c) Immunofluorescence analysis of the location of mitochondrion and lysosome in H9c2 cells. (d) The production of ATP in H9c2 cells determined by ELISA. ##p < 0.01 IR+Ad-PLK1 *vs* IR+Ad-NC, **p < 0.01 sham+Ad-NC vs IR+Ad-NC

The inhibition of PLK1 can repress the mitophagy of breast cancer cells [12]. PLK1 also participates in the repair of myocardium [13]. Therefore, the expression of mitophagy-related proteins was detected by western blotting to investigate the influence of PLK1 on mitophagy in ischemia reperfusion induced myocardial infarction model and H9c2 cells. The results showed that the expression of LC3 II/LC3I, LC3 II and Beclin1 was increased in ischemia reperfusion myocardium and H9c2 cells after the overexpression of PLK1 (Figure 4a and Figure 4b). Next, the location of mitochondria and lysosome in H9c2 cells was determined with the immunofluorescence. Results (Figure 4c) showed that the mitochondria–lysosome fusion gradually happened in H9c2 cells suffered from the hypoxia and reoxygenation stimulation, which implied the occurrence of the mitophagy. The ELISA results (Figure 4d) also showed that the production of ATP in cells was decreased by hypoxia and reoxygenation stimulation, which was rescued by the overexpression of PLK1 in H9c2 cells.

### Overexpression of polo-like kinase 1 (PLK1) activated the expression of p-AMPK in H9c2 cells

**Figure 5.**
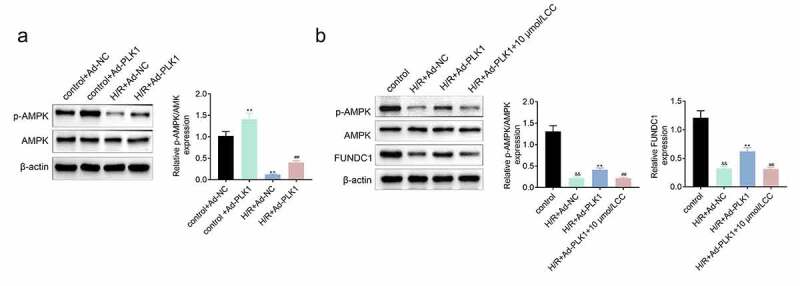
**Overexpression of PLK1 activated the expression of p-AMPK in H9c2 cells**. (a) Western blot analysis of the expression of p-AMPK and AMPK in H9c2 cells. (b) Western blot analysis of the levels of p-AMPK and FUNDC1 in H9c2 cells. ##p < 0.01 H/R+ Ad-PLK1 vs H/R+ Ad-NC, &&p < 0.01 H/R+ Ad-NC vs control, **p < 0.01 H/R+ Ad-PLK1 + 10 μmol/L CC vs H/R+ Ad-PLK1

Some work revealed that the expression of PLK1 activated the expression of p-AMPK, which protected the myocardium from injury [14]. Therefore, the expression of p-AMPK in H9c2 cells stimulated with hypoxia and reoxygenation was detected and the results (Figure 5a) showed that the expression of p-AMPK was enhanced by the overexpression of PLK1 in H/R plus Ad-PLK1 group compared to H/R plus NC group. FUNDC1 is a mitochondrial receptor involved in cardio protection, and AMPK can promote the expression of FUNDC1 [18]. PLK1 overexpression also increased FUNDC1 expression in the H9c2 cells suffered from hypoxia and reoxygenation stimulation. However, the promotional effect of PLK1 on p-AMPK and FUNDC1 expression was suppressed by inhibitor of AMPK (Dorsomorphin) (Figure 5b).

### Inhibition of FUNDC1 further suppressed the mitophagy of H9c2 cells

**Figure 6.**
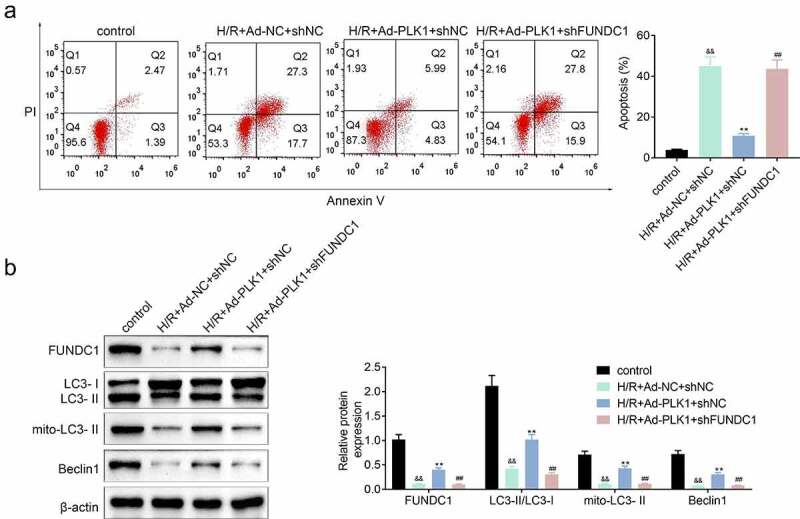
**Inhibition of FUNDC1 abolished the effect of PLK1 overexpression on the mitophagy of H9c2 cells**. (a) flow cytometry analysis of the apoptosis of H9c2 cells . (b) Western blot analysis of the expression of mitophagy related proteins in H9c2 cells. ##p < 0.01 H/R+ Ad-PLK1+ shFUNDC1 vs H/R+ Ad-PLK1+ shNC, &&p < 0.01 H/R+ Ad-NC+shNC vs control, **p < 0.01 H/R+ Ad-NC+shNC vs H/R+ Ad-PLK1+ shNC

The expression of FUNDC1 was inhibited in H9c2 cells transfected with PLK1adenovirusand, the apoptosis of cells was detected. Results (Figure 6a) showed that the suppression of FUNDC1 counteracted the inhibitory effect of PLK1 on hypoxia and reoxygenation induced H9c2 cells apoptosis. Furthermore, the PLK1 overexpression enhanced expression of mitophagy-related protein such as LC3II/LC3I, mito LC3 II and Beclin1 in H9c2 cells was also decreased by the silence of FUNDC1 (Figure 6b).

## Discussion

Myocardial infarction is one of the critical reasons for the occurrence and development of diverse types of heart diseases [30]. Ischemia-reperfusion can induce a variety of tissue damage, including nerve tissues and liver tissues [31]. The heart is also highly sensitive to ischemia and hypoxia [32]. For example, the occurrence of ischemia and hypoxia was able to induce the death of myocardial cells and cause irreversible damage to the heart [33]. Mitochondrial dysfunction induced by ischemia reperfusion was the main reason of the death of myocardial cells [34]. Whereas the moderate mitophagy can protect the myocardium from the ischemia reperfusion injury [35]. Therefore, we speculated that the induction of mitophagy of myocardial cells could relieved the ischemia reperfusion induced damage of myocardium.

PLK1 plays a role in regulating the cell cycle and cell division [36]. In addition, PLK1 can suppress the apoptosis of glioblastoma cells [37]. PLK1 also participates in the DNA damage repair in different cells [38]. Some reports demonstrated that suppression of PLK1 inhibited the mitophagy of breast cancer cells [12]. While, enhancing the expression of PLK1 promoted the proliferation of myocardial cells [39]. In this study, we found that the expression of PLK1 in myocardium of rats was inhibited after the rats suffered from ischemia reperfusion. However, overexpression of PLK1 relieved the ischemia reperfusion induced myocardial infarction and myocardial apoptosis in rats. Enhanced expression of PLK1 also alleviated the apoptosis of H9c2 cells suffered from the hypoxia and reoxygenation stimulation. These results indicated that PLK1 protected the myocardium from suffering the ischemia reperfusion induced damage to myocardium. In addition, the expression of mitophagy-related proteins mito LC3 II and Beclin1 was inhibited in myocardium of ischemia reperfusion model and H9c2 cells, which was rescued by overexpression of PLK1. Moreover, we found that mitochondria were gradually engulfed by lysosomes in H9c2 cells after the overexpression of PLK1. These results suggested that PLK1 protected the H9c2 cells from ischemia reperfusion induced damage by activating the mitophagy.

Furthermore, the activating of p-AMPK can relieve the myocardial ischemia-reperfusion injury of rats through improving the mitochondrial energy metabolism [40,41]. Some work revealed that the PLK1 enhanced the mitosis of cells by promoting the expression of p-AMPK [42]. In this research, we also found that hypoxia and reoxygenation suppressed the expression of p-AMPK in H9c2 cells, which was reversed by overexpression of PLK1. FUNDC1 is a receptor that can regulate mitochondrial function of cells [43]. FUNDC1 was reported to be capable of relieving the symptoms of heart diseases [44]. The FUNDC1 plays a role in protecting the mitochondrion through activating the mitochondrial activity and inducing the mitophagy [45]. Consistently, our results also showed that the inhibition of FUNDC1 suppressed the expression of p-AMPK and mitophagy related proteins in H9c2 cells, which indicated that PLK1 protected the H9c2 cells from hypoxia and reoxygenation by promoting the expression of p-AMPK and FUNDC1.

Above all, we determined the efficacy of PLK1 on the ischemia reperfusion induced myocardial damage of rats. The results of our study revealed that PLK1 protected the ischemia reperfusion induced myocardial damage of rats by inducing the mitophagy and promoting the expression of p-AMPK and FUNDC1. Furthermore, the conclusion of our study could also provide the new therapy for the clinic treatment of heart ischemia reperfusion injury.

## Conclusion

In conclusion, PLK1 alleviated the ischemia reperfusion induced myocardial damage by inducing the mitophagy. In addition, PLK1 induced the mitophagy mainly through activating the p-AMPK/FUNDC1 signaling pathway, which provides a potential new target for treatment of ischemia reperfusion induced myocardial damage.

## Highlight

1. PLK1 relieved the ischemia reperfusion induced myocardial damage by activating mitophagy.

2. PLK1 induced the mitophagy though enhancing the expression of FUNDC1 in H9c2 cells.

3. PLK1 promoted the expression of FUNDC1 by activating the p-AMPK.
